# Neurological manifestations of ehrlichiosis among a cohort of patients: prevalence and clinical symptoms

**DOI:** 10.1186/s12879-024-09607-3

**Published:** 2024-07-17

**Authors:** Osahon Iyamu, Emily J. Ciccone, Abigail Schulz, Julia Sung, Haley Abernathy, Aidin Alejo, Katherine Tyrlik, Victor Arahirwa, Odai Mansour, Dana Giandomenico, Monica M. Diaz, Ross M. Boyce

**Affiliations:** 1 College of Arts and Sciences, University of North Carolina at Chapel Hill, NC 27599 Chapel Hill, USA; 2 Perelman School of Medicine, University of Pennsylvania, PA 19104 Philadelphia, USA; 3 Institute for Global Health and Infectious Diseases, University of North Carolina at Chapel Hill, NC 27599 Chapel Hill, USA; 4 Department of Epidemiology, Gillings School of Global Public Health, University of North Carolina at Chapel Hill, NC 27599 Chapel Hill, USA; 5 College of Medicine, University of Illinois, IL 61605 Peoria, USA; 6 School of Medicine, University of North Carolina at Chapel Hill, NC 27599 Chapel Hill, USA; 7 Department of Neurology, University of North Carolina at Chapel Hill, NC 27599 Chapel Hill, USA; 8 Division of Infectious Diseases, University of North Carolina at Chapel Hill, 123 West Franklin Street, Suite 2151, NC 27516 Chapel Hill, USA

**Keywords:** Ehrlichiosis, Ticks, Neurology

## Abstract

**Background:**

Ehrlichiosis is a potentially fatal tick-borne disease that can progress to involve the central nervous system (CNS) (i.e., neuro-ehrlichiosis), particularly in cases where diagnosis and treatment are delayed. Despite a six-fold national increase in the incidence of ehrlichiosis over the past 20 years, recent data on the prevalence and manifestations of neuro-ehrlichiosis are lacking.

**Methods:**

We conducted a retrospective chart review of all patients tested for ehrlichiosis at University of North Carolina Health facilities between 2018 and 2021 and identified patients who met epidemiological criteria for ehrlichiosis as established by the Council of State and Territorial Epidemiologists and employed by the Centers for Disease Control and Prevention. We estimated the prevalence of neurological symptoms and described the spectrum of neurological manifestations in acute ehrlichiosis, documenting select patient cases in more detail in a case series.

**Results:**

Out of 55 patients with confirmed or probable ehrlichiosis, five patients (9.1%) had neurologic symptoms, which is notably lower than previous estimates. Neurological presentations were highly variable and included confusion, amnesia, seizures, focal neurological deficits mimicking ischemic vascular events, and an isolated cranial nerve palsy, though all patients had unremarkable neuroimaging at time of presentation. All but one patient had risk factors for severe ehrlichiosis (i.e., older age, immunosuppression).

**Conclusions:**

Neuro-ehrlichiosis may lack unifying patterns in clinical presentation that would otherwise aid in diagnosis. Clinicians should maintain a high index of suspicion for neuro-ehrlichiosis in patients with acute febrile illness, diverse neurological symptoms, and negative neuroimaging in lone star tick endemic regions.

## Key points

Ehrlichiosis can progress to involve the central nervous system but may present without unifying patterns of neurological symptoms. Increased awareness of the broad spectrum of clinical presentations in neuro-ehrlichiosis is imperative to facilitate prompt diagnosis and treatment.

## Introduction

Ehrlichiosis is a potentially fatal tick-borne disease classically associated with infection with *Ehrlichia chaffeensis*, an obligate intracellular bacterium that infects monocytes and is transmitted via the lone star tick (*Amblyomma americanum*) [1, 2]. Historically, the lone star tick was primarily confined to the southeastern and midwestern United States (US), though it has undergone rapid geographic expansion as far north as Michigan and the New England region [3, 4]. The six-fold increase in *Ehrlichia* infections in the US over the past 20 years has further established ehrlichiosis as a public health threat [1]. While the case fatality rate for ehrlichiosis is roughly 1% in the general population, national surveillance data indicates that mortality can rise to nearly 15% in children under 5 years of age and over 50% in adults over 70 years old [1, 5]. 

Infection with *E. chaffeensis*, or related species such as *E. ewingii* and less commonly the *Ixodes scapularis*-transmitted *E. muris eauclarensis*, can result in a wide range of symptoms. Most frequently, patients will present with an acute febrile illness accompanied by headache, myalgia, fatigue, and gastrointestinal symptoms; a maculopapular rash may also be present [6, 7]. Leukopenia, thrombocytopenia, anemia, hyponatremia, and elevated liver transaminases are also notable features of ehrlichiosis [2, 7]. While molecular assays are increasingly utilized, indirect immunofluorescence assay (IFA) remains the most common method for diagnosing *Ehrlichia* infection. The diagnosis is confirmed by a four-fold rise in serum immunoglobulin G (IgG) between acute and convalescent samples over a period of 2–4 weeks [8]. However, as serum IgG levels may be undetectable in early illness, relying on a single negative IFA during acute infection can cause ehrlichiosis to be excluded prematurely [9]. 

In cases where diagnosis and treatment are delayed, ehrlichiosis can progress to involve nearly any organ system, including the central nervous system (CNS) and the peripheral nervous system [7]. Ehrlichiosis with neurological symptoms, or neuro-ehrlichiosis, can have a myriad of neurological manifestations, including encephalopathy, meningitis, seizures, ataxia, plexopathies, polyneuropathies, and cranial nerve deficits [10–13]. Despite the relatively severe nature of neurological involvement, there remains a paucity of primary literature describing the incidence, clinical manifestations, and outcomes of neuro-ehrlichiosis. Therefore, the objective of this retrospective cohort study and case series is to describe the prevalence of neurological symptoms in patients with ehrlichiosis and highlight the common clinical manifestations in these patients.

## Materials and methods

The study employed a cross-sectional design that included all individuals tested for ehrlichiosis at University of North Carolina (UNC) Health facilities between March 2018 and February 2021. UNC Health is the largest academic health system in North Carolina, comprised of 12 hospitals and approximately 350 outpatient clinics located across the state. Specifically, we requested medical record numbers for all patients who had undergone diagnostic testing for ehrlichiosis by either IFA or polymerase chain reaction (PCR) from the Carolina Data Warehouse for Health, a central repository containing clinical, research, and administrative data sourced from UNC Health. We then abstracted demographic information, clinical histories, and laboratory results via chart review from the electronic medical record into an electronic database (REDCap) [14]. 

Individuals were considered an ehrlichiosis case if they met one of three diagnostic criteria: (i) a single, acute IgG antibody reciprocal titer of ≥ 1:256, (ii) a four-fold increase in reciprocal titer results between acute and convalescent sera, or (ii) non-reactive acute result with subsequent reactive (i.e., titer ≥ 1:64) convalescent result. By setting the criteria for a single IgG titer at ≥ 1:256, we attempted to increase specificity given the high background rate of seropositivity in the region [15–17]. Each case was then classified as “confirmed” or “probable” based on definitions established by the Council of State and Territorial Epidemiologists (Table 1) [18]. 

**Table 1:** Ehrlichiosis Case definitions as established by the Council of State and Territorial Epidemiologists[18]

Confirmed Case	Probable Case
Meets clinical evidence and laboratory confirmed criteria	Meets clinical evidence and laboratory supportive criteria
**Clinical Evidence**	
One or more of the following: ● Fever ● Headache ● Myalgia ● Anemia ● Leukopenia ● Thrombocytopenia ● Hepatic transaminase elevation	
**Laboratory Confirmed**	**Laboratory Supportive**
Serological evidence of a fourfold change in IgG-specific antibody titer to *E. chaffeensis* antigen by indirect immunofluorescence assay (IFA) between paired serum samples **OR** Detection of *E. chaffeensis* DNA in a clinical specimen via amplification of a specific target by polymerase chain reaction (PCR) assay	Serological evidence of elevated IgG or IgM antibody reactive with *E. chaffeensis* antigen by IFA, enzyme-linked immunosorbent assay (ELISA), dot-ELISA, or assays in other formats* **OR** Identification of morulae in the cytoplasm of monocytes or macrophages by microscopic examination.

*CDC uses an IFA IgG cutoff of > 1:64 and does not use IgM test results independently as diagnostic support criteria.

To estimate the prevalence of neurological symptoms, we performed a detailed review of the medical records of confirmed and probable cases, including examination of clinic notes for outpatient encounters and daily progress notes and discharge summaries for inpatient encounters. Assessment of neurological manifestations potentially related to ehrlichiosis was performed by two investigators (OI, EC), one of whom is board-certified in infectious diseases. Disagreements were adjudicated by another physician with experience managing tick-borne infections (RB). Neurological symptoms were defined as signs and/or symptoms of the following diagnoses: meningitis/encephalitis (e.g. fever, headache, altered mental status, photophobia, phonophobia, neck stiffness), altered mental status (e.g., cognitive impairment, dysarthria, confusion, somnolence, or memory issues), peripheral neuropathy or mononeuropathy, encephalopathy, cranial nerve palsies, brachial plexopathy, and/or seizures. Cases experiencing only headaches or dizziness were recorded in a separate category. Results of brain imaging (computed tomography (CT) scan, magnetic resonance imaging (MRI)) and/or lumbar puncture, if performed, were documented.

During the investigation, two incident cases meeting inclusion criteria occurring outside the pre-specified time period were brought to the attention of the study team. These were included in the case series but were not incorporated into prevalence estimates.

## Results

### Screening and Prevalence

**Fig. 1:**
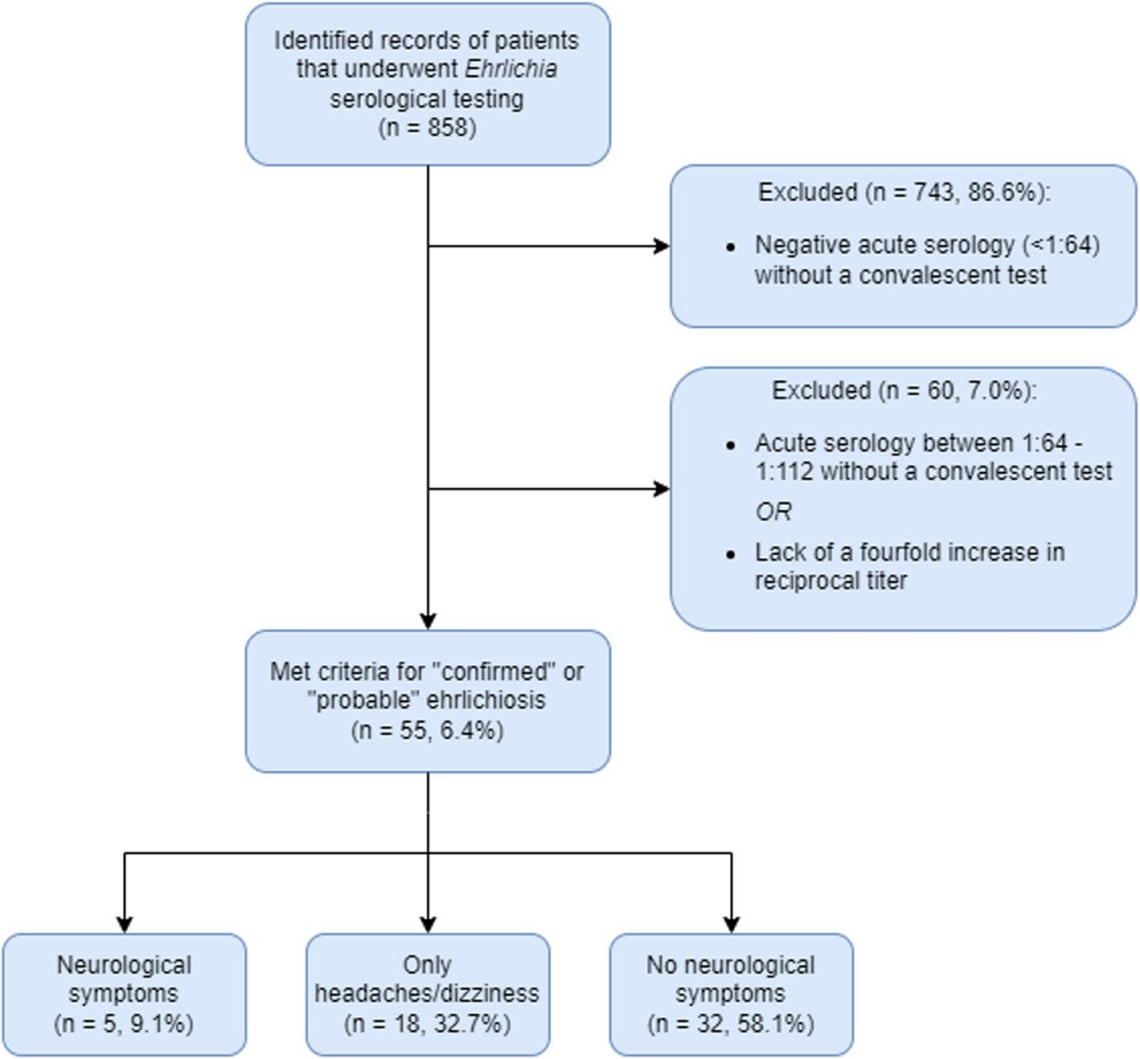
Flowchart for Subject Eligibility Criteria. The parent cohort included 858 individuals who underwent testing for tick-borne diseases. 55 individuals met the criteria for this sub-study

A total of 858 medical records of individuals tested for *Ehrlichia* were identified between March 2018 and February 2021. Of these, 743 records were excluded due to non-reactive acute *Ehrlichia* serologies (< 1:64) with no convalescent testing performed. An additional 60 records were excluded due to a single acute serology < 1:256 with no convalescent testing performed or lack of a four-fold increase in reciprocal titer. 55 individuals met criteria for probable or confirmed ehrlichiosis and were subsequently included in the analysis (Fig. 1). Review of the clinical documentation identified 5 (9.1%) subjects with evidence of neurologic complications, all of whom met criteria for probable ehrlichiosis. Four of the five patients presented with altered mental status and were subsequently admitted to the hospital. Three of the cases are discussed in more detail below, as well as the two cases identified outside of the study’s time frame. Table 2 summarizes the clinical, laboratory, and imaging results for all seven patients.

**Table 2:** Summary of demographic, clinical, and laboratory information for patients with ehrlichiosis presenting with neurologic symptoms

	Patient 1	Patient 2	Patient 3	Patient 4	Patient 5	Add’l Patient 1	Add’l Patient 2
Demographic							
Age	42	71	83	85	86	66	56
Sex	M	M	F	F	F	F	F
Race	White	White	White	White	White	White	White
**Signs & Symptoms**							
Headache		X			X	X	X
Fever		X	X			X	X
AMS / Encephalopathy		X	X	X	X	X	X
Dysarthria		X					
Memory loss			X				
Seizures		X					
Cranial Nerve Palsies	X						
**Laboratory**							
CSF Analysis							
Nucleated cells	-	40	-	-	-	-	18
Protein	-	195	-	-	-	-	70
Glucose	-	121	-	-	-	-	74
Culture	-	Negative	-	-	-	-	Negative
Ehrlichia IgG							
Acute	1:512	> 1:1024	1:512	1:256	1:512	1:64	1:128
Convalescent	1:512	-	-	-	-	1:256	> 1:1024
SFGR IgG							
Acute	< 1:64	< 1:64	1:128	< 1:64	1:512	< 1:64	1:64
Convalescent	-	-	-	-	-	-	1:128
**Imaging**							
Abnormal CT	-	Yes	-	No	No	-	No
Abnormal MRI	No	Yes	-	-	No	-	No
**Clinical Course**							
Length of symptoms prior to care seeking (days)	47	4	1	3	7	8	7
Length of inpatient stay (days)	-	27	1	3	2	-	10
Length of doxycycline treatment (days)	14	10	14	10	10	10	14
Response to doxycycline	Yes	No	Partial	Yes	Yes	Yes	Yes
Clinical outcome	Recovered	Discharged to hospice	Died of myocardial infarction 4 months later	Recovered	Recovered	Recovered	Recovered

*AMS = Altered mental status. CSF = Cerebrospinal fluid. SFGR = Spotted Fever Group Rickettsioses*

### Case presentations

#### Patient 1

A 42-year-old white male presented to an urgent care clinic with one day of right ear fullness and acute hearing loss. On examination, his right external ear and tympanic membrane were normal, but air conduction was greater than bone conduction on the right (Rinne’s test) suggestive of sensorineural hearing loss. He was given a preliminary diagnosis of “sudden sensorineural hearing loss,” prescribed prednisone 60 mg daily for 10 days, and referred to audiology and otolaryngology. An audiological evaluation one week later was remarkable for moderate low-frequency sensorineural hearing loss of the right ear, likely due to right cranial nerve VIII involvement. MRI of the brain without contrast did not show any abnormalities. Subsequently, the patient, who lives in a county with one of the highest incidences of ehrlichiosis in the state [19], recalled a tick bite on his buttocks approximately 2 weeks before symptom onset. Serological testing for Lyme disease and spotted fever group Rickettsia (SFGR) was non-reactive, but *Ehrlichia* IgG by IFA was reactive at a reciprocal titer of 1:512. He was started on a 14-day course of doxycycline – approximately 34 days after symptom onset - with rapid improvement in symptoms. Convalescent testing sent 66 days after symptom onset returned at a similar titer of 1:512. Following completion of treatment, his symptoms resolved.

### Patient 4

An 85-year-old white female with a history of hypertension, dyslipidemia, and non-Hodgkin’s lymphoma in remission presented to her primary care clinic with five days of fatigue, unsteadiness, and anorexia. Two weeks prior, she had removed a tick from her right groin. On examination in clinic, she was notably afebrile but had an irregular heart rhythm and a small, firm, hyperpigmented papule at the site of the tick bite. She was initiated on doxycycline. Laboratory testing demonstrated thrombocytopenia (98 × 10^9^ cells/L), hyponatremia (128 mmol/L), acute kidney injury (creatinine 2.5 mg/dL) and elevations in aspartate aminotransferase (170 U/L) and alanine aminotransferase (93 U/L), all of which were new compared to two weeks prior. She was instructed to present to the emergency department (ED), which she did the following day. In the ED, her daughter reported that the patient was experiencing a new expressive aphasia and was frequently repeating herself. She was admitted to the hospital, where she became progressively disoriented. A non-contrast CT scan of the head did not show any acute abnormalities. In addition to doxycycline, she received intravenous fluids to correct her hyponatremia. By hospital day 3, her clinical and neurological status had returned to baseline, and she was discharged to complete a 10-day course of doxycycline. The results of tick-borne disease testing revealed a reactive *Ehrlichia* IgG IFA (1:256) and non-reactive SFGR serology. Lyme serology was not performed. At outpatient follow-up one week later, she still reported some fatigue but generally felt back to baseline. Repeat laboratory testing showed resolution of her thrombocytopenia, acute kidney injury, and liver inflammation.

### Patient 5

An 86-year-old white female with no known medical history was brought to the ED by her family for five days of headache, chills, nausea, and vomiting after her granddaughter found the patient confused and unable to recall the names of family members. In the ED, she was afebrile with vital signs notable for a blood pressure of 196/94 mmHg. She was oriented only to person, but not time or place. She had multiple macular areas of erythema on her arms, which she attributed to working in the garden. Laboratory testing demonstrated mild hyponatremia (131 mmol/L); a complete blood count, urinalysis, and testing for SARS-CoV-2 were unremarkable. A plain film of the chest and non-contrast CT of the head did not reveal any abnormalities. She was admitted to the hospital and, given her outdoor exposures, was initiated on empirical doxycycline for tick-borne diseases. A non-contrast MRI of the brain identified areas suggestive of “mild chronic small vessel ischemic disease” but no other abnormalities. Further neurological and psychiatric examination revealed impaired cognition and judgment, amnesia, acalculia, and finger agnosia. Based on these findings, she was diagnosed with Gerstmann syndrome, a condition caused by a lesion of the posterior parietal lobe of the dominant hemisphere and characterized by a tetrad of acalculia, finger agnosia, agraphia, and right-left disorientation [20]. Her neurological status rapidly improved, and she was discharged on hospital day 3 to complete the course of doxycycline. Results of serological testing for tick-borne disease returned with reactive titers for both *Ehrlichia* and SFGR at 1:512 and non-reactive titers for Lyme.

### Additional cases

#### Additional patient 1

A 66-year-old white female with a history of hypertension presented to a primary care clinic eight days after developing chills, fevers to 101 °F, nausea, vomiting, diarrhea, anorexia, fatigue, arthralgias, headache, dizziness, blurry vision, impaired coordination, and word-finding difficulties while on a cruise in Germany. She lived in a rural area and reported a possible tick bite six days prior to symptom onset. Laboratory studies were notable for mild leukocytosis (11.4 × 10^9^ cells/L) and slightly elevated total bilirubin (1.5 mg/dL). Her symptoms persisted, so she re-presented to her primary care doctor 12 days into illness, at which point tick-borne disease testing was performed. She was not prescribed treatment at this time but began taking veterinary doxycycline that she acquired from a friend. Her *Ehrlichia* titer returned positive at 1:64 (Lyme and RSMF titers were negative), and she was referred to the infectious diseases clinic. Her symptoms improved with doxycycline over the subsequent 19 days, and upon evaluation in the infectious diseases clinic, only some occasional nausea and fatigue persisted. The doxycycline therapy was stopped. A repeat *Ehrlichia* titer returned at 1:256. She went on to have complete resolution of symptoms.

### Additional patient 2

A 56-year-old white female with a history of non-insulin dependent type 2 diabetes, rheumatoid arthritis on upadacitinib, and cirrhosis due to non-alcoholic fatty liver disease presented to the ED with a one-week history of fevers, cough, and myalgias. The patient lived in a rural area, where she was an avid gardener and went on frequent hikes. Upon arrival, she was febrile and tachycardic, and she was subsequently admitted for sepsis. Exam at the time was unremarkable, and she was noted to be alert and oriented and without lymphadenopathy or rash. Labs were notable for a new anemia (hemoglobin 10.9 g/dL), thrombocytopenia (41 × 10^9^ cells/L), hyponatremia (123 mmol/L), and an elevated aspartate aminotransferase and alanine aminotransferase (93 and 97 U/L, respectively). A plain film of the chest was unremarkable. Testing for SARS-CoV-2 and a multiplex respiratory pathogen panel were negative. Blood cultures were obtained, and she was started on empiric broad spectrum IV antibiotics with vancomycin and cefepime as well as doxycycline. Her immunosuppressants were held.

Despite this treatment, she became progressively more obtunded, requiring intubation for airway protection on hospital day 3. IV ampicillin and acyclovir were initiated, and cerebrospinal fluid (CSF) from a lumbar puncture showed a lymphocytic pleocytosis of 18 cells/µl, elevated protein of 70 mg/dL, and normal glucose of 74 mg/dL. Further CSF studies were negative, including bacterial culture, HSV PCR, enterovirus PCR, HHV-6 PCR, cryptococcal antigen, and an arbovirus antibody panel. MRI of the brain with and without contrast was unremarkable. As blood cultures returned negative, acyclovir, vancomycin, ampicillin, and cefepime were discontinued. Acute sera for *Ehrlichia* and SFGR IgG were sent and returned at 1:128 and 1:64, respectively. *Ehrlichia* PCR from the blood returned positive, and doxycycline was continued. She defervesced by hospital day 4. Her mental status began to improve, allowing for extubation on day 4 and eventual discharge to acute rehabilitation on hospital day 10. At follow-up four weeks after her initial presentation, her mental status had returned to baseline without residual neurologic deficits. Convalescent sera showed an *Ehrlichia* IgG titer of ≥ 1:1024. SFGR convalescent sera was 1:128.

## Discussion

In this review of individuals that underwent testing for ehrlichiosis at a large academic medical system in North Carolina between 2018 and 2021, 9.1% (5 of 55) of those with confirmed or probable ehrlichiosis had neurological symptoms. While encephalopathy was a common finding, particularly in elderly individuals with more severe disease, we also observed more varied manifestations including an isolated cranial nerve palsy. Notably, acute focal neurological deficits mimicking ischemic vascular events were seen in two patients, including a presentation compatible with Gerstmann syndrome, which is classically associated with an infarct to the dominant parietal lobe [20]. The majority of patients had unremarkable brain imaging, an observation consistent with prior descriptions of neuro-ehrlichiosis [10, 21]. Taken together with previous literature, our case series highlights the varied neurological presentations in ehrlichiosis [13, 22–24] and provides further evidence that neuro-ehrlichiosis may lack unifying clinical patterns or findings on neuroimaging that facilitate diagnosis. Instead, providers must maintain a high level of suspicion in patients with a broad range of neurological presentations, including encephalopathy, especially during spring and summer months when human-tick encounters are common.

One area where our study diverges from previous literature is the frequency of neurological symptoms in ehrlichiosis. Specifically, our prevalence estimate is substantially lower than previous reports (Table 3) [25–28]. While the method of laboratory confirmation varied (i.e., PCR, IFA on paired sera, single IFA on unpaired sera), these studies found that over 20% of individuals with ehrlichiosis had neurological symptoms. A number of factors may explain this difference. First, the most recent of these studies was conducted nearly two decades ago. During the interval period, the incidence of *Ehrlichia* infection in the US has increased more than six-fold [1], likely attributable not only to increased transmission, but also increased awareness of *Ehrlichia* among clinicians, contributing to more testing and earlier identification of infections. In addition to increasing the detection of milder cases of ehrlichiosis, a heightened clinical suspicion may have led to earlier initiation of doxycycline, an intervention that has been shown to reduce the number of patients that progress to a more severe disease with neurological manifestations [29, 30]. 

**Table 3:** Summary of previous studies reporting neurological manifestations of ehrlichiosis

Study	Time Period	Location	Sample	Method of Confirmation	Hospitalized (%)	Reported Manifestations of CNS Disease (%)
Eng et al., 1990 [28]	1988	US, nationwide	40 patients undergoing serological testing for RMSF at state health department or private laboratories	Paired IFA with four-fold change in titers	85	Confusion [29] Meningitis [11]
Fishbein et al., 1994 [27]	1985–1990	US, nationwide	237 patients undergoing serological testing for RMSF at state health department or private laboratories	Paired IFA with four-fold change in titers	61	Confusion [20] Ataxia (< 10) Hearing loss (< 10) Meningitis (< 10) Seizures (< 10) Stupor (< 10)
Everett et al., 1994 [25]	1989–1992	Missouri	30 patients seeking care at an academic and Veterans Affairs hospital and affiliated clinics	Positive PCR *or* Paired IFA with four-fold change in titers	77	Mental status changes [20] Photophobia [13]
Olano et al., 2003 [26]	1997–1998	US, nationwide	41 patients identified by a national reference laboratory	Single IFA with IgG titers ≥ 1:64 *or* Single IFA with IgM titers ≥ 1:20	56	Confusion [22] Hallucinations [10] Stupor [7] Meningitis [7] Coma [2] Seizures [2]

*CNS = Central nervous system. US = United States. RMSF = Rocky Mountain Spotted Fever. IFA = Immunofluorescence assay. PCR = Polymerase chain reaction*

It is also possible that our study captured cases of *E. ewingii* ehrlichiosis, which has not only risen in incidence since first being described in 1999 [1, 31], but often results in milder illness and fewer severe complications compared to *E. chaffeensis* [32, 33]. While nationwide studies estimate that infection with *E. ewingii* occurs in 2–9% of patients with ehrlichiosis, [31, 34] routine surveillance of *E. ewingii* is complicated by cross-reactivity on IFA with other *Ehrlichia* species such as *E. chaffeensis*, as well as lack of widespread availability of *E. ewingii*-specific PCR tests [31]. Interestingly, the seroprevalence of *E. ewingii* may actually be higher than *E. chaffeensis* among *A. americanum* populations in some endemic states, including North Carolina [35–37]. Further human and entomological studies using species-specific methods such as PCR are needed to fully quantify the contribution of *E. ewingii* to overall ehrlichiosis cases in our region.

With the exception of one patient, individuals in our cohort with neurological symptoms also had risk factors for severe ehrlichiosis (i.e., older age or receiving immunosuppressive therapy). Similarly, some patients also had clinical or radiographic evidence of chronic microvascular disease, some of which may have been caused by prior, unrecognized ischemic events. These patients were likely not only predisposed to severe disease, but also more likely to experience an exacerbation or recrudescence of underlying neurological disorders. It is also probable that the older adults in our cohort had some degree of underlying cerebral small vessel disease that predisposed them to neurological symptoms. However, the prompt neurologic recovery in the stroke-like deficits observed in patients 4 and 5 after receiving doxycycline strongly suggests that infection itself, rather than an ischemic process, was the primary driver.

Our study has a number of strengths including the large catchment area of the UNC Health system, the multi-year study period, rigorous inclusion criteria that ensured a relatively high specificity for acute ehrlichiosis, and a greater number of identified *Ehrlichia* cases as compared to most previous studies [25–28]. 

Our study also has important limitations. Foremost among these is the retrospective design and reliance on routine medical records to evaluate for neurological symptoms. Some patients may have experienced subtle manifestations that were not identified and documented, which could have resulted in an underestimate of the prevalence. For example, we excluded 18 patients from our analysis who presented with headache and/or dizziness alone due to the non-specific nature of these symptoms, but it is possible that with further investigation, including CSF analysis, we would have identified more cases. Given that CSF analysis and neuroimaging were normal or absent in several patients, we are not able to definitively attribute the neurologic manifestations observed to true CNS infection by *Ehrlichia*, particularly given the presence of risk factors such as older age, multiple comorbidities, and concomitant electrolyte derangements in our cohort. Additionally, with the majority of patients described in our series not undergoing lumbar puncture, the possibility of a viral co-infection also cannot be excluded. For instance, patients in our cohort were not tested for Heartland virus, which is transmitted by *A. americanum* and is known to cause altered mental status, though the extraordinarily low prevalence of this virus in *A. americanum* populations in the southeastern US makes co-infection with *Ehrlichia* and Heartland virus unlikely [38–40]. The rapid response to appropriate antibiotic therapy observed in five out of seven of our cases was also more consistent with a bacterial, rather than viral infection. Overall, these limitations illustrate the importance of obtaining CSF studies and neuroimaging (i.e., MRI) in patients with suspected CNS infection, particularly when the etiology of neurological symptoms is unclear.

Furthermore, most cases were diagnosed using serological methods with few demonstrating a four-fold rise in titers between acute and convalescent sera. While this adds an element of uncertainty to the diagnosis, by utilizing higher cutoff titers in our inclusion criteria, we strove to minimize the number of false positives that arose from relying on a single acute titer for diagnosis [15, 17]. It is also likely that reliance on serological diagnosis resulted in underestimation of prevalence as false negative serology in the first week of illness is frequently identified, and only about 10% of patients in our cohort underwent convalescent testing [17, 41]. Fortunately, our institution is moving towards molecular diagnosis, which eliminates the challenge of patient follow-up for convalescent testing. We also cannot completely rule out *Rickettsia* species involvement in some cases given the coincident reactivity of SFGR IgG. However, the reactive titer more likely reflects exposure to a nonpathogenic SFGR, namely *Rickettsia amblyommatis*, which is the most prevalent *Rickettsia* species in *A. americanum* ticks in the region and may be a common co-exposure in ehrlichiosis [35, 42]. Finally, only one of the 7 patients had an electroencephalogram performed to rule out seizures or post-ictal state as a cause of the neurologic findings.

## Conclusions

Our study found that neurological manifestations occurred in approximately 9% of patients with ehrlichiosis presenting to a large academic health system in North Carolina. Patients in our series demonstrated a wide variety of neurological manifestations without clear patterns or commonalities in the neurologic deficits experienced. However, older age or immunosuppression were shared features in those presenting with neurological symptoms. This study highlights the need to consider neuro-ehrlichiosis in patients who present with acute febrile illness, neurologic deficits, and unrevealing neuroimaging, particularly in regions where the lone star tick is endemic.

## Data Availability

No datasets were generated or analysed during the current study.
